# Synchronous Haemorrhagic Tumors of the Anterior Maxilla and Oropharynx

**DOI:** 10.1002/ccr3.72602

**Published:** 2026-04-26

**Authors:** Brian Maloney, Blessing Obasi, Jason Byrne, Mary Collins, Conor Bowe, Róisín O'Connor

**Affiliations:** ^1^ National Maxillofacial Unit St James's Hospital Dublin Ireland; ^2^ Histopathology Department St James's Hospital Dublin Ireland; ^3^ Division of Oral and Maxillofacial Surgery, Medicine, Pathology and Radiology Dublin Dental University Hospital Trinity College Dublin Dublin Ireland

**Keywords:** angiosarcoma, epithelioid, immunohistochemistry, metastases, sarcoma

## Abstract

Oral epithelioid angiosarcoma arising in the oral cavity is exceptionally rare. This case reports the first instance of synchronous primary epithelioid angiosarcoma in the oral cavity and oropharynx. Accurate diagnosis requires an understanding of the importance of immunohistochemistry to differentiate this vascular tumour from similar pathologic entities.

## Graphical abstract

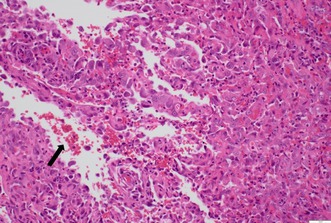

## 1 Introduction

Angiosarcoma is a highly aggressive malignant tumor arising from vascular mesenchymal tissue, characterized by its marked potential for locoregional recurrence and distant metastases [1]. Despite the ubiquitous presence of endothelium throughout the body, angiosarcoma remains exceedingly rare, accounting for only 2% of all cases of soft tissue sarcoma [1].

Histologically, angiosarcoma is categorized into three morphological subtypes: the angiomatous pattern with epithelioid features, the spindle cell pattern, and the solid pattern [2]. The epithelioid subtype, first described by Weiss et al., is a particularly aggressive variant defined by solid and sheet‐like growth of malignant endothelial cells with epithelioid morphology [3]. Although less common than other subtypes, epithelioid angiosarcoma poses significant diagnostic challenges due to its overlapping features with other epithelioid neoplasms, including carcinoma, melanoma, and epithelioid sarcoma [4].

To date, only eight cases of primary epithelioid angiosarcoma in the oral cavity have been reported (Table 1) [12, 13, 14, 15, 16, 17, 18, 19]. To the authors' best knowledge, this is the first reported case of synchronous tumors of this elusive entity and only the second reported case of epithelioid angiosarcoma involving the tonsils. We herein present this rare case, highlighting the myriad of histologic and clinical differential diagnoses that were considered, the range of histological analyses employed, and the eventual medical and surgical management of the case.

**TABLE 1:** Previously documented cases of primary oral epithelioid angiosarcoma.

Author	Age	Sex	Location	Clinical course	Therapy	Metastases/recurrence	Markers
Freedman and Kerpal., 1992 [5]	32	M	Maxilla	No evidence of disease at 18 months	Surgery	N/A	VIII +ve
Sasaki et al., 1996 [6]	69	M	Hard palate	Died at 9 months	Surgery and adjuvant chemo‐radiotherapy	Stomach, vertebra and cerebrum	CC31, VIII, Cytokeratin +ve EMA ‐ve
Triantafillidou et al., 2002 [7]	50	F	Maxilla	No disease at 3 years	Surgery	Nil	VIII +ve
Favia et al., 2002 [8]	74	F	Hard palate	Died at 3 months	Surgery and adjuvant chemo‐radiotherapy	N/A	CD31, CKAE1/3 + ve CD34, VIII ‐ve
Agaimy et al., 2012^29^	71	F	Right tonsil	Metastases to GI tract	Surgery	Intraoral and GI	CD31, VIII, CKAE1/AE3+
Nagata et al., 2014 [9]	55	M	Mandibular gingiva	Died at 9 months	Surgery	Thoracic, and vertebral	CD34, EMA, CKAE1/AE3 + ve
Komatsu et al., 2020 [10]	66	M	Mandibular gingiva	No disease at 2 years	Surgery	Nil	CD31, VIII +ve Cytokeratin ‐ve
El Ouazzani et al., 2023 [11]	73	M	Mandibular gingiva	Recurrence and died at 2 months	Surgery	Yes	CD31, ERG, CKAE1/3, CK7 + ve CD34, EMA ‐ve
Current case	70	F	Maxillary gingiva and left oropharynx	Died at 6 months	Radical radiotherapy only	Nil	MNF116, CD31, ERG +ve, HHV‐8, S100, SMA, EMA, CD34 ‐ve

## 2 Case History/Examination

A 70‐year‐old female self‐presented to the Dublin Dental University Hospital Emergency Department with an enlarging painless mass on the anterior maxillary gingiva, which had begun several months prior. There was significant growth of the mass over the past few months, which prompted the patient to present to the emergency department. The evolving mass had been treated with multiple courses of oral antibiotics by the patient's dentist, to no avail. The patient had a background of hypertension and osteoarthritis. She was a current smoker and had a 30‐pack‐year history.

**FIGURE 1:**
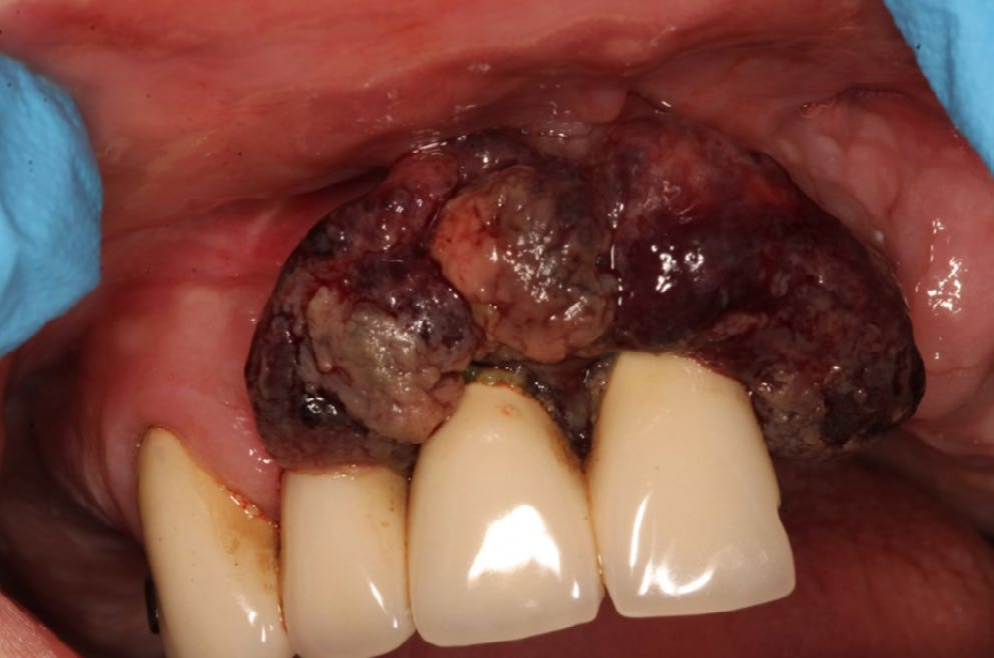
Pre‐operative appearance of a lobulated, vascular mass enveloping maxillary teeth and gingiva.

**FIGURE 2:**
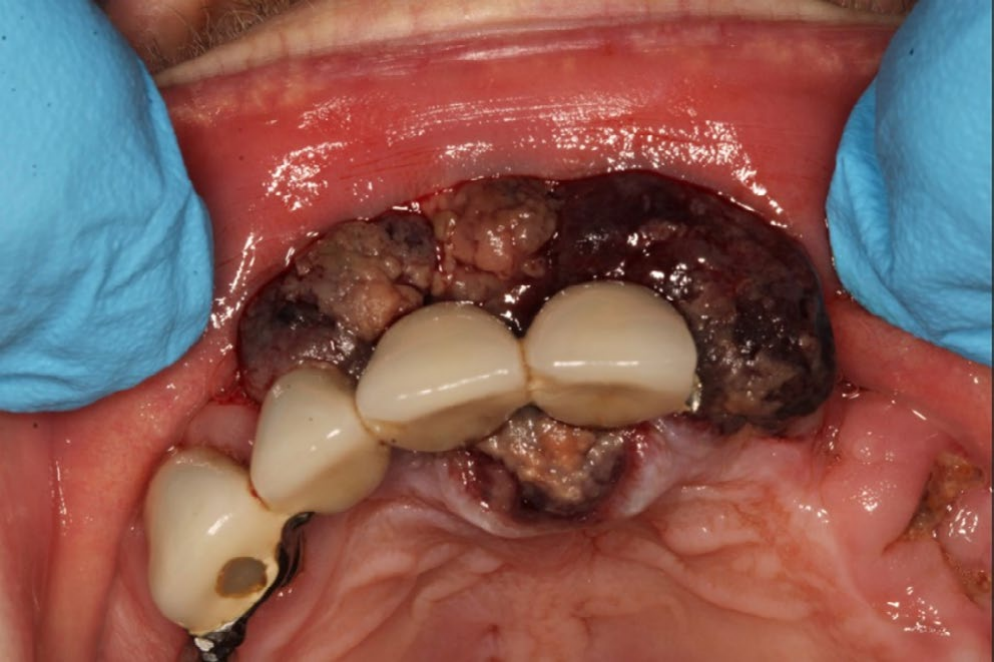
Pre‐operative appearance of a lobulated, vascular mass enveloping maxillary teeth and gingiva.

On clinical examination extra‐orally, there was no cervical lymphadenopathy. Intra‐orally, there was a 30 × 15 mm exophytic haemorrhagic friable mass on the alveolar gingiva of the maxilla anteriorly, enveloping the teeth and extending onto the palatal mucosa (Figures 1 and 2). The left tonsil also appeared enlarged with haemorrhagic vesicles.

The patient was planned for a conservative excisional biopsy of the alveolar gingival mass under intravenous sedation with removal of the remaining anterior maxillary teeth and bridgework.

## 3 Differential Diagnosis

The maxillary alveolar mass was clinically highly suspicious for malignancy as it was enveloping teeth and extending onto the palatal mucosa. At this juncture, one must have an open and yet focused mind regarding the myriad potential differential diagnoses. Diagnoses that are commonly encountered in the oral cavity must be considered, along with rarer tumors and indeed metastatic tumors, which can be well disguised and lead to significant diagnostic difficulty.

Kaposi sarcoma (KS) is a locally aggressive malignant vascular neoplasm associated with human herpesvirus 8 (HHV‐8). It is the most common tumor diagnosed in patients with human immunodeficiency virus (HIV)/acquired immunodeficiency syndrome (AIDS) [20]. The oral cavity, especially the hard palate and gingiva, is a common site for AIDS‐associated KS in the head and neck [20]. KS can have a variable presentation, in the form of erythematous patches in the early stage of the disease, with progression to multifocal, confluent masses over time [20]. The exophytic mass in our case would have been clinically consistent with a presentation of KS. Most noteworthy, while considering this differential, is that our patient did not have a history of HIV/AIDS.

Angiosarcoma is an aggressive vascular neoplasm. More than 50% of cases occur in cutaneous sites in the form of multifocal papules, while soft tissue angiosarcoma generally arises within the deep muscles of the lower extremities [21]. The latter form can also demonstrate bone and hepatic involvement [22, 23]. The etiology of angiosarcoma is largely unknown; it is more common in males, and incidence peaks in the seventh decade [22]. Angiosarcoma arising in the head and neck region usually presents with rapidly growing, sometimes painful, haemorrhagic masses [22]. Our patient did not complain of pain; however, she did report that the mass had been growing over the last several months. The clinical appearance of a haemorrhagic mass would be consistent with the classic presentation of angiosarcoma at other common anatomical sites. Therefore, this entity was considered unlikely given its rarity in the oral cavity [23].

Metastatic involvement of the oral cavity reflects secondary tumor deposition arising from a malignancy originating at a distant extrinsic site. This group represents approximately 1% of oral malignancies, with the gingiva being the most common soft tissue location, accounting for more than 50% of cases, followed by the tongue as the second most common site in 25% of cases [24, 25]. The most common metastatic tumors to the oral cavity in females are from breast, genitourinary, and colorectal tumors [26]. Carcinomas, including adenocarcinomas, are the most frequent types of metastatic tumors. Metastatic sarcomas to the oral cavity are extremely rare [25]. Immunohistochemistry is a valuable tool in distinguishing metastatic disease from primary oral malignancy and elucidating the tissue of origin [25]. Our patient did not have a known history of malignancy, and therefore a thorough history and clinical examination would have been crucial at presentation. In addition, as part of the multidisciplinary workup, the presence of a primary distant tumor can often be elucidated through Positron Emission Tomography (PET) with Computed Tomography (CT) [27].

Mucosal melanoma (MM) is rare in the head and neck region, and, despite MM representing less than 1% of all melanomas, most oral cavity cases arise in the maxillary gingiva and palate, in the exact location where this mass was clinically located [28]. MM, as with cutaneous melanoma, can wear many guises and can present as a macule or a nodular, pigmented, amelanotic or haemorrhagic mass [29]. MM usually presents in older patients and incidence peaks in the seventh decade; therefore, our patient would have been in the correct age cohort to be considered slightly higher risk. The molecular risk factors for MM are rather different to those for cutaneous and uveal melanoma, with higher rates of KIT mutations and also, evidently, MM lacks the ultraviolet radiation signature [30].

Oral lymphomas are the third most common malignancy in the oral cavity; however, they are overall rare and account for only 3% of all lymphomas [28]. Most head and neck lymphomas arise in the Waldeyer ring and head and neck lymph nodes. Among extra‐nodal lymphomas, the non‐Hodgkin variant is by far the most common subtype [31]. Diffuse large B‐cell lymphoma is the most common type of NHL and accounts for most oral lymphoma cases [28]. Clinically, oral lymphoma can mimic other aggressive malignancies due to its rapid growth, vascular appearance, propensity for bleeding, and its mass‐forming nature [5]. Immunohistochemistry plays a critical role in distinguishing lymphoma from other malignancies. While primary gingival haematolymphoid neoplasms are exceptionally rare, they must be considered in the differential diagnosis of any rapidly growing and clinically aggressive neoplasm in the oral cavity [31].

Pyogenic granuloma, also known as lobular capillary haemangioma, is a benign, non‐neoplastic lesion that often represents a reactive overgrowth of capillary loops. Clinically, it typically presents as a painless, polypoid growth with secondary surface ulceration and bleeding, showing a predilection for the anterior maxillary gingiva and a propensity for recurrence after excision [6]. Despite it being a benign entity, it has been included in the differential diagnosis as its clinical appearance can be nodular and haemorrhagic, similar to the current case. Additionally, it can grow rapidly and bleed easily, which raises concern for a neoplastic process.

Oral squamous cell carcinoma (OSCC) is, by far, the most common oral cavity malignancy [28]. It is a malignant epithelial neoplasm that arises from the oral mucosa, generally in pre‐existing dysplastic epithelium [28]. Oral subsite involvement varies, with the tongue and floor of the mouth being the most common sites affected [8]. Risk factors for developing OSCC included tobacco (both smoked and smokeless), alcohol use and *Areca catechu* palm nut use, in all its many guises [7]. Clinically, in the aforementioned case, the gingival mass was haemorrhagic in nature, which would not be the classic macroscopic appearance of OSCC. The patient had known risk factors for OSCC, and the gingiva is a known location where OSCC can arise, although not commonly. Therefore, OSCC must be considered in the primary instance, common things being common [8].

Haemangioendothelioma (HE) is a low‐grade angiocentric vascular neoplasm [32]. The clinical and biological behavior of HE is intermediate between angiosarcoma and haemangioma [28]. Reported cases of intraoral HE are rare and tend to affect younger individuals [9]. The presentation of HE in the oral cavity is commonly a solitary, painless lump, most often affecting the lips, tongue, buccal mucosa, and palate [9]. While HE was unlikely in the above case owing to the patient's age, this low‐grade vascular neoplasm, in particular the epithelioid variant (EHE), can mimic other vascular tumors owing to the overlapping clinical features of reddish appearance, bleeding and sometimes ulceration [9]. TBC An important distinguishing feature between haemangioendothelioma and malignant entities is the less aggressive behavior of the former entity. Owing to the overlapping clinical and histological features with other vascular tumors, however, careful histopathological and immunochemical evaluation is key in distinguishing these entities.

## 4 Conclusion and Results

**FIGURE 3:**
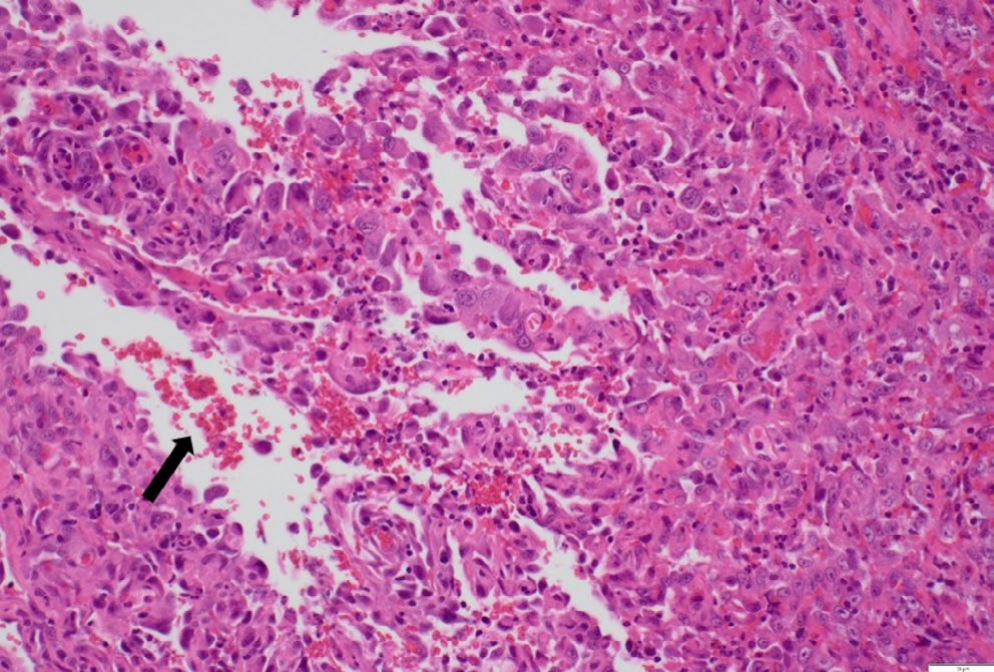
Photomicrograph of a low‐power view of partially ulcerated squamous mucosa with underlying vascular proliferation showing anastomosing vascular channels. Hematoxylin and eosin stain. Magnification ×20.

**FIGURE 4:**
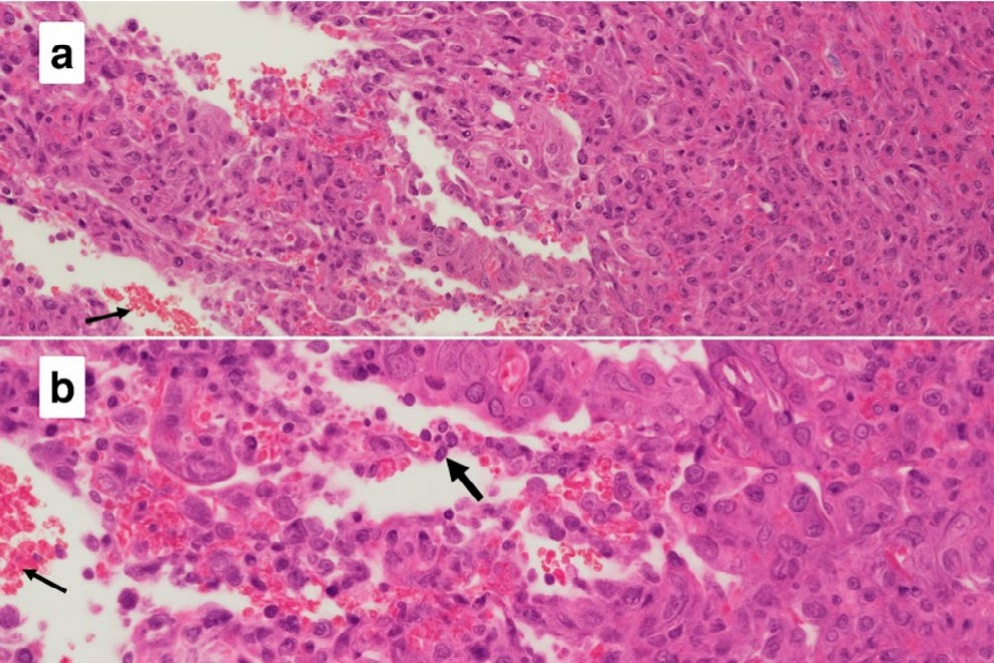
Composite photomicrograph showing (a) malignant epithelioid cells forming vascular channels with associated extravasated red blood cells (narrow arrow) and (b) evidence of hob‐nailing (thick arrow). Hematoxylin and eosin stain. Magnification: (a) ×00 (b) ×400.

**FIGURE 5:**
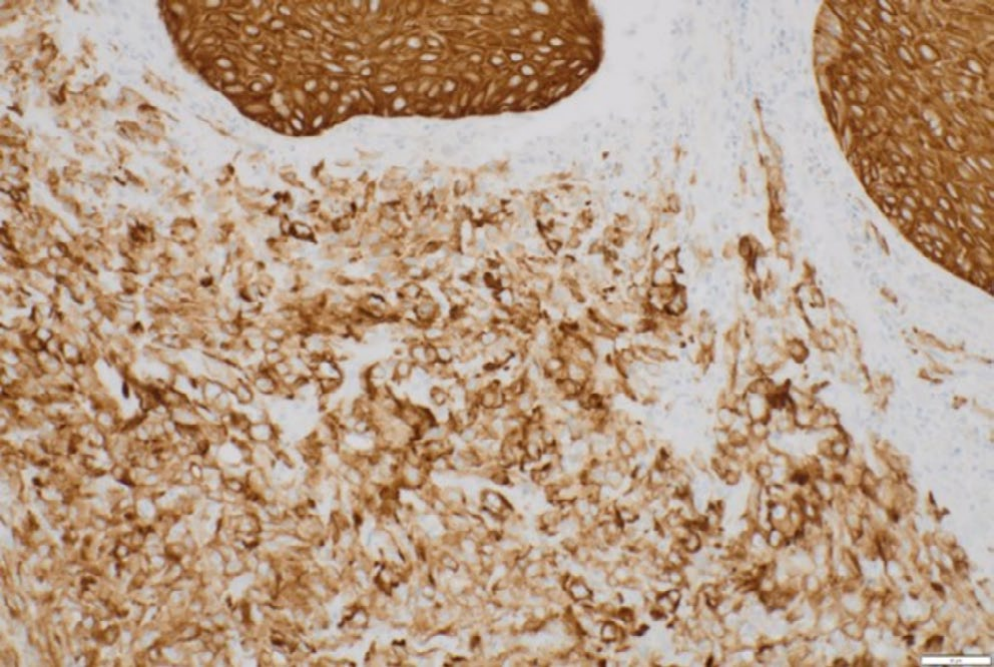
Photomicrograph showing diffuse expression of MNF116. Immunohistochemical stain. Magnification ×200.

**FIGURE 6:**
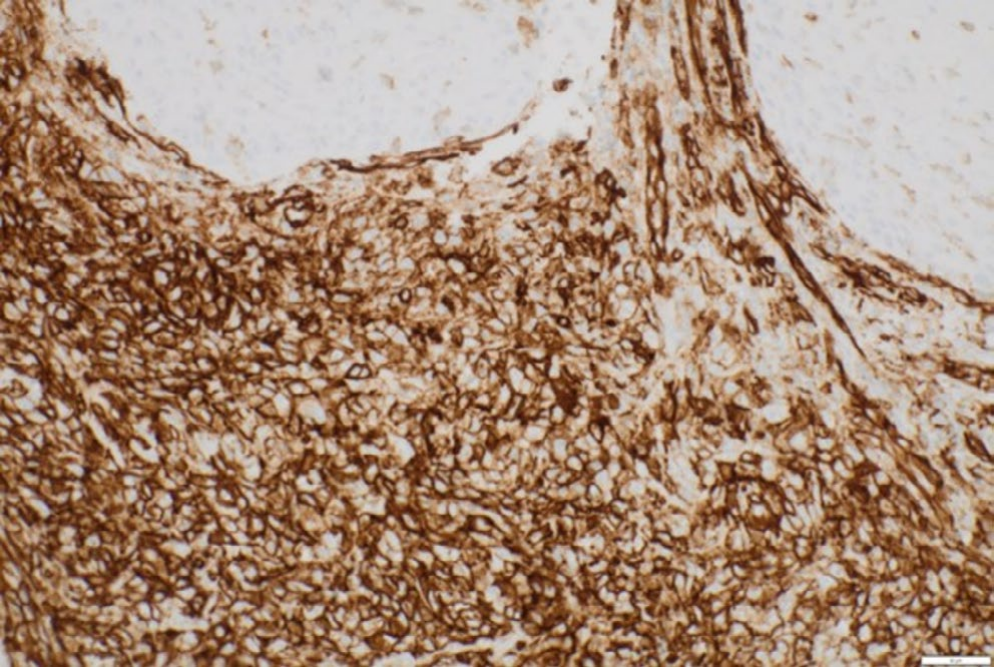
Photomicrograph magnification showing strong and diffuse cytoplasmic expression of CD31 in the neoplastic cells. Immunohistochemical stain.

**FIGURE 7:**
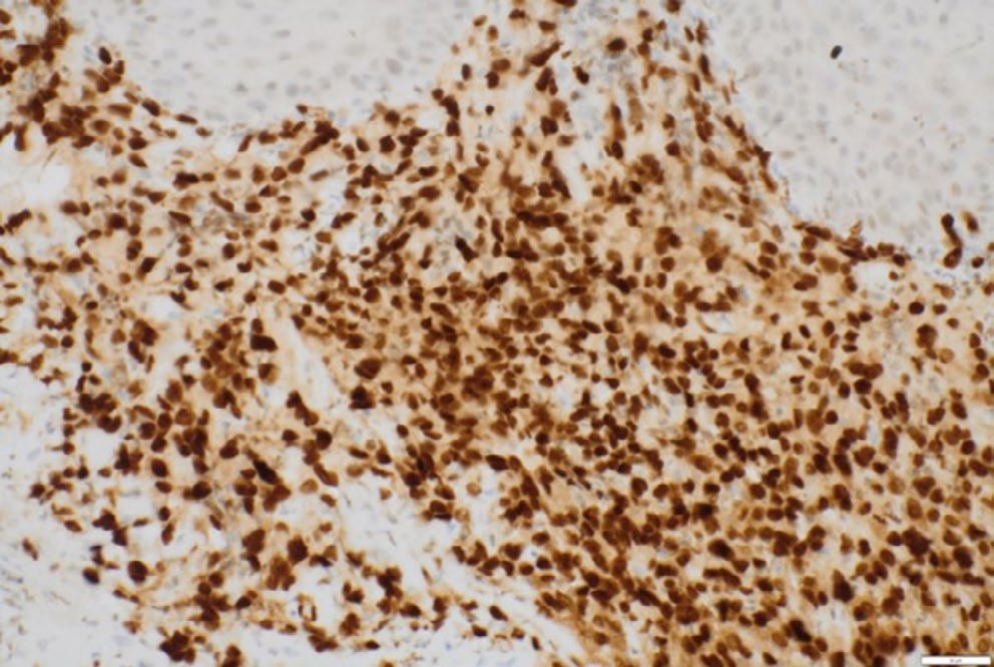
Photomicrograph showing a strong and diffuse nuclear reaction for ERG in the neoplastic cells. Immunohistochemical stain. Magnification ×200.

Histology demonstrated partially ulcerated squamous mucosa overlying a proliferation of malignant polygonal epithelioid cells with abundant eosinophilic cytoplasm forming anastomosing vascular channels, with hob‐nailing, brisk mitoses, focal necrosis and extravasated red blood cells (Figures 3 and 4). The nuclei were enlarged, vesicular, and pleomorphic with irregular nuclear contours and prominent nucleoli. On immunohistochemistry, the neoplastic cells were positive for broad‐spectrum cytokeratin marker MNF116 and endothelial markers CD31 and ERG (Figures 5, 6, 7), consistent with epithelioid angiosarcoma, and were negative for HHV‐8, S100, SMA, EMA, and CD34.

**FIGURE 8:**
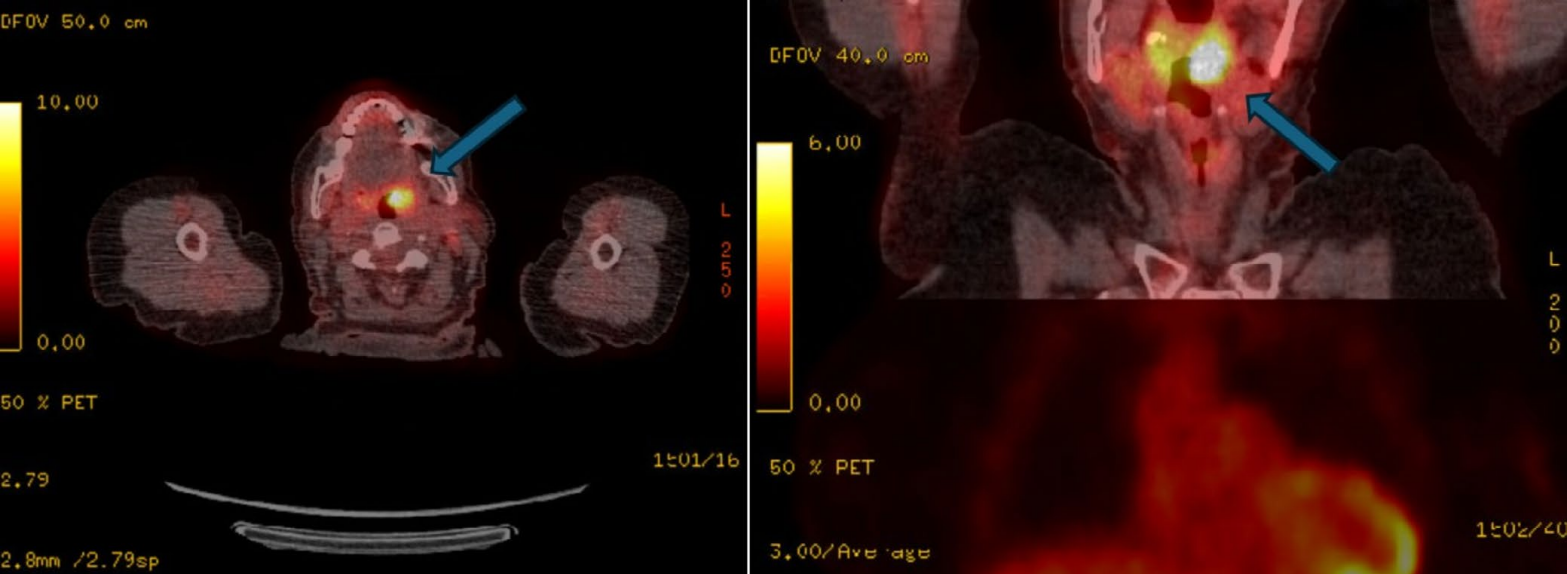
Staging PET‐CT showing axial and coronal views with fluorodeoxyglucose (FDG) uptake in the left tonsillar region.

The unexpected diagnosis of this rare sarcoma required onward referral of the patient to the National Maxillofacial Surgery Unit in St James's Hospital, Dublin. Workup imaging for the patient (CT facial bones, MRI neck, PET‐CT) revealed an intensely FDG‐avid 2.1 × 1.2 × 3.1 cm left oropharyngeal tonsillar mass, suspicious for primary malignant tumor and avid left level Ib, II, and III nodes consistent with ipsilateral nodal metastases (Figure 8). MRI also revealed a 9 mm left level IIa lymph node. There was an additional finding of a 6 mm fluorodeoxyglucose FDG‐avid lesion in the superficial lobe of the right parotid gland, which was proven to be a Warthin tumor on a needle biopsy. No residual tumor was identified in the anterior maxilla.

The patient underwent an examination under anesthesia and pan‐endoscopy to facilitate an incisional biopsy of the left tonsil. The histology revealed squamous mucosa undermined by a malignant proliferation of epithelioid cells, which were forming vascular channels, together with extravasated red blood cells and haemosiderin deposition. The specimen had similar histomorphological and immunohistochemical features to the previous biopsy of the anterior maxilla, thus confirming a synchronous epithelioid angiosarcoma in the left tonsil.

The patient was discussed at the sarcoma multidisciplinary meeting in St. Vincent's University Hospital. The outcome from this meeting was radical radiotherapy and no further surgery. During the course of radiotherapy, the patient required medical admission to St James's Hospital due to hypotension, pneumonia and reduced oral intake. She required gastrostomy insertion during this time. About 1 year from the initial presentation, the patient suffered multifocal haemorrhagic strokes and acute posterior circulation multifocal infarcts with haemorrhagic transformation and hydrocephalus. She was transferred to the care of palliative medicine and passed away shortly after.

## 5 Discussion

Angiosarcoma exhibits a roughly equal distribution between the sexes and can occur at any age, although it is most commonly diagnosed in older adults [10]. It may involve any visceral organ or soft tissue, but certain anatomical sites are more frequently affected. According to Young et al., the head and neck region is the most commonly involved site (27%), followed by the breast (20%), lower extremities (15%), and trunk (10%) [22]. Approximately 12% of all cases of angiosarcoma present with an unknown primary origin. Within the head and neck, cutaneous lesions predominate and represent nearly 50% of all tumors in this region [23].

In contrast, involvement of the oral cavity is distinctly uncommon. Fanburg‐Smith et al. reported that oral cavity angiosarcoma constitutes only 1% of all angiosarcoma cases [23]. Furthermore, primary oral cavity angiosarcoma, defined by the absence of metastatic spread from a distant site, is exceedingly rare, with most reported cases being secondary in origin [11]. When angiosarcoma occurs in the oral cavity, both as primary and secondary disease, it can affect various sites, including the tongue, gingiva, palate, and parotid gland. The maxilla is more commonly affected than the mandible [23].

As described by Hart and Mandavalli, epithelioid angiosarcoma is characterized by an infiltrative sheet of malignant endothelial cells with marked proliferative activity, frequent intratumoral hemorrhage, necrosis and surface ulceration [4]. The vascular channels are tortuous and lined with epithelioid endothelial cells with eosinophilic cytoplasm. Nuclei are pleomorphic, hyperchromatic and vesicular with prominent macronuclei. Mitotic activity is brisk [4]. Current consensus from the American Joint Committee on Cancer and the College of American Pathologists is that histologic grading is not recommended for angiosarcoma. This is because conventional grading systems correlate poorly with prognosis [33]. This was concluded in the case series of oral angiosarcoma carried out by Fanburg‐Smith et al., wherein certain cases with high‐grade disease had superior treatment outcomes compared to those with low‐grade disease [23]. The predictive value of histologic parameters in angiosarcoma has been investigated, but no consensus has been reached [1].

Obtaining a definitive histopathologic diagnosis of epithelioid angiosarcoma is challenging and necessitates the concurrent application of immunohistochemical techniques with a panel of specific endothelial markers to differentiate epithelioid angiosarcoma from morphologically similar entities [23]. Ultimately, identifying the vascular phenotype is essential for accurate diagnosis [34]. Epithelioid angiosarcoma typically exhibits strong, diffuse staining for vascular markers CD31 [4, 35]. Other markers, such as FLI1, Factor VIII, D2‐40, SMA, and CD34, show variable expression, with the latter two often being negative, as was the case in our patient. Importantly, a subset of epithelioid angiosarcoma cases may show cytokeratin positivity, which can mimic poorly differentiated carcinoma and lead to diagnostic confusion [35]. Therefore, integration of histological and immunophenotypic data alongside clinical correlation is essential for accurate diagnosis.

The distinctive histological features of KS help in differentiating it from other entities. There are characteristic features of cytoplasmic hyaline globules, spindled cells with distinctive borders, and plasma cells are absent [36]. In addition, HHV‐8 is a consistently positive marker in KS [37]. Melanoma can be excluded based on the presence of subtle histologic differences, an absence of staining for vascular markers, and positivity for melanocytic markers (Table 2) [41]. Cytokeratin positivity and negative staining for endothelial markers are usually useful features in metastatic carcinoma. However, this feature is not ubiquitous, as some poorly differentiated carcinomas are cytokeratin‐negative [38]. Benign entities can be differentiated from epithelioid angiosarcoma readily based on the presence of histologic features: vasoformative foci, infiltrative growth and cellular atypia.

**TABLE 2:** Immunohistochemical profiles of differentials for angiosarcoma.

Entity	Positive IHC markers	Negative IHC markers	
Oral epithelioid angiosarcoma	Vimentin, CD31, ERG, FLI‐1, Factor VIII, CD34, cytokeratin	S100, HMB‐45, Melan‐A, CD45, Desmin, high molecular weight CK, CAMTA1	Endothelial panel (CD31, ERG) is diagnostic. CK can mislead towards carcinoma [23].
Metastatic carcinoma	Pan‐CK, CAM5.2, EMA	CD31, CD34, Factor VIII, FLI‐1, ERG	The presence of epithelial CK positivity and lack of endothelial markers differentiates it from AS [38].
Kaposi Sarcoma	CD31, CD34, HHV‐8	High molecular weight CK, S100, HMB‐45	Endothelial markers overlap; HHV‐8 positivity is distinguishing [37].
Endothelial haemangioendothelioma	CD31, CD34, FLI‐1, Factor VIII, Pan CKCAMTA1	S100, HMB‐45, CD45	Less cytologic atypia, does not demonstrate an aggressive infiltrative growth pattern [39].
Haematolymphoid neoplasm	CD45 (haematopoeitic origin), B‐cell (CD19, CD2), T‐cell (CD3, CD5, CD7), NK‐cell (CD56), myeloid markers (CD13, CD33)	Cytokeratins (AE1/AE3, CK20), S100, Melan‐A, Desmin, SMA, Vimentin	Lymphoid marker positivity is distinctive for specific subtypes [40].
Melanoma	S100, HMB‐45, Melan‐A	CD31, CD34, Factor VIII, FLI‐1, ERG	Melanocytic markers are definitive for melanoma [41].

Haematolymphoid neoplasms and angiosarcoma can exhibit overlapping histological features, creating potential diagnostic ambiguity. While both groups can exhibit high‐grade features of infiltration, with cellular atypia and increased proliferation, the growth architecture of these entities differs [11]. Angiosarcoma demonstrates characteristic irregular anastomosing vascular channels, while haematolymphoid neoplasms often form nodular, follicular patterns [4, 11]. Owing to these similarities, histologic assessment must be combined with immunohistochemistry to define lineage.

Haematolymphoid neoplasms can be distinguished from non‐haematolymphoid malignancies by their lack of epithelial, melanocytic, and mesenchymal marker expression [42]. The integration of specific lymphoid markers for haematolymphoid neoplasms is essential in distinguishing these malignancies [42]. CD45 confirms hematopoietic origin, while lineage can be further defined with additional B cell markers (CD20, CD79A), T cell markers (CD3, CD5, CD7, CD4, CD8), NK cell marker (CD56), and myeloid markers (CD13) [40, 42]. Additional markers exist for further subtyping, such as CD30, Cyclin D1, MUM‐1, CD10, and BCL‐6 [42]. Table 2 highlights the characteristics of IHC profiles and distinguishing histological features for the aforementioned entities.

The aforementioned differential of epithelioid haemangioendothelioma is more challenging to rule out. Epithelioid haemangioendothelioma has a similar immunohistochemical profile, with positivity for vascular markers, namely CD34, CD31, and factor VIII [32]. However, epithelioid haemangioendothelioma often demonstrates a more lobulated growth pattern compared to the destructive pattern seen in angiosarcoma. Cellular atypia is less common in epithelioid haemangioendothelioma, and it demonstrates a lower mitotic rate. In addition, this entity rarely demonstrates necrosis [32]. CAMTA1 positivity on IHC in epithelioid haemangioendothelioma may also be useful, as the epithelioid subtype is defined by recurrent WWTR1‐CAMTA1 gene fusion in 90% of cases [43].

Therapeutic management of angiosarcoma remains challenging due to its rarity and biological heterogeneity [44]. While primary surgical excision with clear margins, combined with radiotherapy pre‐ or post‐operatively, offers the best chance for long‐term survival, this approach is feasible only when the tumor is clinically resectable [15]. There is currently no universally accepted treatment algorithm, and evidence‐based guidance remains limited due to the low incidence of individual angiosarcoma subtypes. The British Sarcoma Group has recently issued best practice recommendations based on expert consensus, which suggest potential roles for chemotherapy, tyrosine kinase inhibitors, immunotherapy, and other biologic agents, in select cases [45].

In general, the prognosis for angiosarcoma in the head and neck is poor [46]. These tumors are highly prone to both local recurrence and distant metastases, commonly involving the lungs, bones and liver. Five‐year overall survival rates are estimated at around 40%, but drop to 15% in the presence of distant disease [1]. Death most often results from the progression of local disease or widespread metastatic dissemination and organ failure [1].

## 6 Conclusion

Angiosarcoma is a highly aggressive malignancy which rarely presents in the oral cavity. The epithelioid variant is even less common. The incidence of primary epithelioid angiosarcoma arising in the oral cavity is exceptionally rare. To the author's best knowledge, this is the first and only documented case of synchronous primary epithelioid angiosarcoma in the oral cavity and oropharynx. The application of immunohistochemical techniques is essential in differentiating epithelioid angiosarcoma from similar pathologic entities. The long‐term prognosis of angiosarcoma remains poor, and future endeavors should aim to improve treatment options for patients.

## Author Contributions

**Brian Maloney:** conceptualization, data curation, visualization, writing – original draft, writing – review and editing. **Blessing Obasi:** data curation, formal analysis. **Jason Byrne:** investigation, resources, visualization, writing – original draft, writing – review and editing. **Mary Collins:** conceptualization, investigation, methodology, writing – review and editing. **Conor Bowe:** investigation, resources, supervision, writing – review and editing. **Róisín O'Connor:** conceptualization, investigation, methodology, resources, supervision, writing – review and editing.

## Funding

The authors have nothing to report.

## Ethics Statement

Written consent was obtained from the patient included in this report.

## Consent

Written consent was provided by the patient in this case to use the case details for research purposes.

## Conflicts of Interest

The authors declare no conflicts of interest.

## Data Availability

Data sharing not applicable to this article as no datasets were generated or analysed during the current study.

## References

[1] D. Buehler , S. R. Rice , J. S. Moody , et al., “Angiosarcoma Outcomes and Prognostic Factors: A 25‐Year Single Institution Experience,” American Journal of Clinical Oncology 37, no. 5 (2014): 473–479, 10.1097/COC.0b013e31827e4e7b. 23428947 PMC3664266

[2] C. D. Fletcher , “Vascular Tumors: An Update With Emphasis on the Diagnosis of Angiosarcoma and Borderline Vascular Neoplasms,” Monographs in Pathology 38 (1996): 181–206. 8744278

[3] W. M. Weiss , T. S. Riles , T. H. Gouge , and H. H. Mizrachi , “Angiosarcoma at the Site of a Dacron Vascular Prosthesis: A Case Report and Literature Review,” Journal of Vascular Surgery 14, no. 1 (1991): 87–91. 1829490

[4] J. Hart and S. Mandavilli , “Epithelioid Angiosarcoma: A Brief Diagnostic Review and Differential Diagnosis,” Archives of Pathology & Laboratory Medicine 135, no. 2 (2011): 268–272, 10.5858/135.2.268. 21284449

[5] V. P. Wagner , C. I. Rodrigues‐Fernandes , M. V. R. Carvalho , et al., “Mantle Cell Lymphoma, Malt Lymphoma, Small Lymphocytic Lymphoma, and Follicular Lymphoma of the Oral Cavity: An Update,” Journal of Oral Pathology & Medicine 50, no. 6 (2021): 622–630, 10.1111/jop.13214. 34101256

[6] R. Kamal , P. Dahiya , and A. Puri , “Oral Pyogenic Granuloma: Various Concepts of Etiopathogenesis,” Journal Oral Maxillofac Pathology 16, no. 1 (2012): 79–82, 10.4103/0973-029X.92978. PMC3303528 22434943

[7] S. Petti , “Lifestyle Risk Factors for Oral Cancer,” Oral Oncology 45, no. 4–5 (2009): 340–350, 10.1016/j.oraloncology.2008.05.018. 18674956

[8] M. D. P. Paglioni , S. A. Khurram , B. I. I. Ruiz , et al., “Clinical Predictors of Malignant Transformation and Recurrence in Oral Potentially Malignant Disorders: A Systematic Review and Meta‐Analysis,” Oral Surgery, Oral Medicine, Oral Pathology, Oral Radiology 134, no. 5 (2022): 573–587, 10.1016/j.oooo.2022.07.006. 36153299

[9] Z. J. Sun , L. Zhang , W. F. Zhang , M. J. Alsharif , X. M. Chen , and Y. F. Zhao , “Epithelioid Hemangioma in the Oral Mucosa: A Clinicopathological Study of Seven Cases and Review of the Literature,” Oral Oncology 42, no. 5 (2006): 441–447, 10.1016/j.oraloncology.2005.07.012. 16266821

[10] M. G. Fury , C. R. Antonescu , K. J. Van Zee , M. E. Brennan , and R. G. Maki , “A 14‐Year Retrospective Review of Angiosarcoma: Clinical Characteristics, Prognostic Factors, and Treatment Outcomes With Surgery and Chemotherapy,” Cancer Journal 11, no. 3 (2005): 241–247, 10.1097/00130404-200505000-00011. 16053668

[11] WHO Classification of Tumours Editorial Board , Soft Tissues and Bone Tumours, vol. 9 (International Agency for Research on Cancer, 2020), https://publications.iarc.who.int/Book‐And‐Report‐Series/Who‐Classification‐Of‐Tumours/Soft‐Tissue‐And‐Bone‐Tumours‐2020.

[12] P. D. Freedman and S. M. Kerpel , “Epithelioid Angiosarcoma of the Maxilla,” Oral Surgery, Oral Medicine, and Oral Pathology 74, no. 3 (1992): 319–325, 10.1016/0030-4220(92)90068-2. 1407994

[13] M. Sasaki , H. Kumamoto , and K. Ooya , “An Autopsy Case of Epithelioid Angiosarcoma of the Maxilla,” Oral Medicine Pathology 1, no. 1 (1996): 38–42, 10.3353/omp.1.38.

[14] G. Favia , L. Lo Muzio , R. Serpico , and E. Maiorano , “Angiosarcoma of the Head and Neck With Intra‐Oral Presentation. A Clinico‐Pathological Study of Four Cases,” Oral Oncology 38, no. 8 (2002): 757–762, 10.1016/S1368-8375(02)00045-3. 12570054

[15] K. Triantafillidou , N. Lazaridis , and T. Zaramboukas , “Epithelioid Angiosarcoma of the Maxillary Sinus and the Maxilla: A Case Report and Review of the Literature,” Oral Surgery, Oral Medicine, Oral Pathology, Oral Radiology, and Endodontology 94, no. 3 (2002): 333–337, 10.1067/moe.2002.126022. 12324789

[16] A. Agaimy , H. Kirsche , S. Semrau , H. Iro , and A. Hartmann , “Cytokeratin‐Positive Epithelioid Angiosarcoma Presenting in the Tonsil: A Diagnostic Challenge,” Human Pathology 43, no. 7 (2012): 1142–1147, 10.1016/j.humpath.2011.10.018. 22406364

[17] M. Nagata , Y. Yoshitake , H. Nakayama , et al., “Angiosarcoma of the Oral Cavity: A Clinicopathological Study and a Review of the Literature,” International Journal of Oral and Maxillofacial Surgery 43, no. 8 (2014): 917–923, 10.1016/j.ijom.2014.02.008. 24656496

[18] Y. Komatsu , I. Miyamoto , Y. Ohashi , et al., “Primary Epithelioid Angiosarcoma Originating From the Mandibular Gingiva: A Case Report of an Extremely Rare Oral Lesion,” World Journal of Surgical Oncology 18, no. 1 (2020): 260, 10.1186/s12957-020-01999-1. 33010804 PMC7533036

[19] H. El Ouazzani , O. Hamidi , A. Habimana , et al., “Primary Epithelioid Angiosarcoma of the Mandibular Gingiva: Diagnostic Pitfalls, About an Unusual Entity,” Journal of Surgical Case Reports 2024, no. 5 (2024): rjae323, 10.1093/jscr/rjae323. 38800505 PMC11126337

[20] M. Fatahzadeh and R. A. Schwartz , “Oral Kaposi's Sarcoma: A Review and Update,” International Journal of Dermatology 52, no. 6 (2013): 666–672, 10.1111/j.1365-4632.2012.05758.x. 23679875

[21] J. M. Meis‐Kindblom and L. G. Kindblom , “Angiosarcoma of Soft Tissue: A Study of 80 Cases,” American Journal of Surgical Pathology 22, no. 6 (1998): 683–697, 10.1097/00000478-199806000-00005. 9630175

[22] R. J. Young , N. J. Brown , M. W. Reed , D. Hughes , and P. J. Woll , “Angiosarcoma,” Lancet Oncology 11, no. 10 (2010): 983–991, 10.1016/S1470-2045(10)70023-1. 20537949

[23] J. C. Fanburg‐Smith , M. A. Furlong , and E. L. B. Childers , “Oral and Salivary Gland Angiosarcoma: A Clinicopathologic Study of 29 Cases,” Modern Pathology 16, no. 3 (2003): 263–271, 10.1097/01.MP.0000056986.08999.FD. 12640107

[24] A. Hirshberg , A. Shnaiderman‐Shapiro , I. Kaplan , and R. Berger , “Metastatic Tumours to the Oral Cavity – Pathogenesis and Analysis of 673 Cases,” Oral Oncology 44, no. 8 (2008): 743–752, 10.1016/j.oraloncology.2007.09.012. 18061527

[25] G. Kumar and B. Manjunatha , “Metastatic Tumors to the Jaws and Oral Cavity,” Journal Oral Maxillofac Pathology 17, no. 1 (2013): 71, 10.4103/0973-029X.110737. PMC3687193 23798834

[26] A. Hirshberg , R. Berger , I. Allon , and I. Kaplan , “Metastatic Tumors to the Jaws and Mouth,” Head and Neck Pathology 8, no. 4 (2014): 463–474, 10.1007/s12105-014-0591-z. 25409855 PMC4245411

[27] R. Ebner , G. T. Sheikh , M. Brendel , J. Ricke , and C. C. Cyran , “ESR Essentials: Staging and Restaging With FDG‐PET/CT in Oncology—Practice Recommendations by the European Society for Hybrid, Molecular and Translational Imaging,” European Radiology 35, no. 4 (2024): 1894–1902, 10.1007/s00330-024-11094-8. 39384589 PMC11914360

[28] WHO Classification of Tumours Editorial Board , Head and Neck Tumours, vol. 9 (International Agency for Research on Cancer, 2023), https://tumourclassification.iarc.who.int/chapters/67.

[29] M. Mihajlovic , S. Vlajkovic , P. Jovanovic , and V. Stefanovic , “Primary Mucosal Melanomas: A Comprehensive Review,” International Journal of Clinical and Experimental Pathology 5, no. 8 (2012): 739–753. 23071856 PMC3466987

[30] K. C. Furlan , D. Saeed‐Vafa , T. M. Mathew , et al., “Utility of UV Signature Mutations in the Diagnostic Assessment of Metastatic Head and Neck Carcinomas of Unknown Primary,” Head and Neck Pathology 18, no. 1 (2024): 11, 10.1007/s12105-024-01620-x. 38393464 PMC10891032

[31] S. Kemp , G. Gallagher , S. Kabani , V. Noonan , and C. O'Hara , “Oral Non‐Hodgkin's Lymphoma: Review of the Literature and World Health Organization Classification With Reference to 40 Cases,” Oral Surgery, Oral Medicine, Oral Pathology, Oral Radiology, and Endodontology 105, no. 2 (2008): 194–201, 10.1016/j.tripleo.2007.02.019. 17604660

[32] D. Ortiz Requena , J. M. Velez‐Torres , J. A. Diaz‐Perez , C. Gomez‐Fernandez , E. A. Montgomery , and A. E. Rosenberg , “Mesenchymal Neoplasms of the Tongue: A Clinicopathologic Study of 93 Cases,” Human Pathology 150 (2024): 42–50, 10.1016/j.humpath.2024.06.005. 38876200

[33] B. P. Rubin , C. R. Antonescu , F. H. Gannon , et al., “Protocol for the Examination of Specimens From Patients With Tumors of Bone,” Archives of Pathology & Laboratory Medicine 134, no. 4 (2010): e1–e7, 10.5858/134.4.e1. 20367293

[34] M. Vermaat , D. Vanel , H. M. Kroon , et al., “Vascular Tumors of Bone: Imaging Findings,” European Journal of Radiology 77, no. 1 (2011): 13–18, 10.1016/j.ejrad.2010.06.052. 20828961

[35] J. Cao , J. Wang , C. He , and M. Fang , “Angiosarcoma: A Review of Diagnosis and Current Treatment,” American Journal of Cancer Research 9, no. 11 (2019): 2303–2313. 31815036 PMC6895451

[36] W. Grayson and L. Pantanowitz , “Histological Variants of Cutaneous Kaposi Sarcoma,” Diagnostic Pathology 3, no. 1 (2008): 31, 10.1186/1746-1596-3-31. 18655700 PMC2526984

[37] F. G. Nunes Rosado , D. M. Itani , C. M. Coffin , and J. M. Cates , “Utility of Immunohistochemical Staining With FLI1, D2‐40, CD31, and CD34 in the Diagnosis of Acquired Immunodeficiency Syndrome–Related and Non–Acquired Immunodeficiency Syndrome‐Related Kaposi Sarcoma,” Archives of Pathology & Laboratory Medicine 136, no. 3 (2012): 301–304, 10.5858/arpa.2011-0213-OA. 22372906

[38] K. Vidyavathi , C. Prasad , M. Harendra Kumar , and R. Deo , “Pseudovascular Adenoid Squamous Cell Carcinoma of Oral Cavity: A Mimicker of Angiosarcoma,” Journal of Oral and Maxillofacial Pathology 16, no. 2 (2012): 288–290, 10.4103/0973-029X.99092. 22923907 PMC3424951

[39] U. Flucke , R. J. Vogels , N. De Saint Aubain Somerhausen , et al., “Epithelioid Hemangioendothelioma: Clinicopathologic, Immunhistochemical, and Molecular Genetic Analysis of 39 Cases,” Diagnostic Pathology 9, no. 1 (2014): 131, 10.1186/1746-1596-9-131. 24986479 PMC4100035

[40] A. L. Weber , A. Rahemtullah , and J. A. Ferry , “Hodgkin and Non‐Hodgkin Lymphoma of the Head and Neck,” Neuroimaging Clinics of North America 13, no. 3 (2003): 371–392, 10.1016/S1052-5149(03)00039-X. 14631680

[41] M. L. Prasad , A. A. Jungbluth , K. Iversen , A. G. Huvos , and K. J. Busam , “Expression of Melanocytic Differentiation Markers in Malignant Melanomas of the Oral and Sinonasal Mucosa: The American Journal of Surgical Pathology,” Diagnostic Pathology 25, no. 6 (2001): 782–787, 10.1097/00000478-200106000-00010. 11395556

[42] J. Cho , “Basic Immunohistochemistry for Lymphoma Diagnosis,” Blood Research 57, no. S1 (2022): S55–S61, 10.5045/br.2022.2022037. PMC9057666 35483927

[43] L. A. Doyle , C. D. M. Fletcher , and J. L. Hornick , “Nuclear Expression of CAMTA1 Distinguishes Epithelioid Hemangioendothelioma From Histologic Mimics,” American Journal of Surgical Pathology 40, no. 1 (2016): 94–102, 10.1097/PAS.0000000000000511. 26414223

[44] S. Atarbashi‐Moghadam , F. Atarbashi‐Moghadam , M. Niazmand , and S. Shahrabi‐Farahani , “Metastatic Sarcomas of the Oral Cavity: A Systematic Review,” Journal of Stomatology, Oral and Maxillofacial Surgery 125, no. 2 (2024): 101656, 10.1016/j.jormas.2023.101656. 38738551

[45] A. J. Hayes , I. F. Nixon , D. C. Strauss , et al., “UK Guidelines for the Management of Soft Tissue Sarcomas,” British Journal of Cancer 132, no. 1 (2025): 11–31, 10.1038/s41416-024-02674-y. 38734790 PMC11724041

[46] W. M. Lydiatt , A. R. Shaha , and J. P. Shah , “Angiosarcoma of the Head and Neck,” American Journal of Surgery 168, no. 5 (1994): 451–454, 10.1016/S0002-9610(05)80097-2. 7977971

